# Comparable effectiveness of 45- and 20-min post-infusion scalp cooling time in preventing paclitaxel-induced alopecia — a randomized controlled trial

**DOI:** 10.1007/s00520-022-07090-7

**Published:** 2022-05-02

**Authors:** Rieneke T. Lugtenberg, Corina J. G. van den Hurk, Carolien H. Smorenburg, Linda Mosch, Danny Houtsma, Margaret A. G. den Hollander-van Deursen, Ad A. Kaptein, Hans Gelderblom, Judith R. Kroep

**Affiliations:** 1 Department of Medical Oncology, Leiden University Medical Center, Albinusdreef 2, P.O. Box 9600, Leiden, 2300 RC Leiden The Netherlands; 2 Department of Research & Development, The Netherlands Comprehensive Cancer Organisation, Utrecht, The Netherlands; 3 Department of Medical Oncology, Antoni Van Leeuwenhoek, The Netherlands Cancer Institute, Amsterdam, The Netherlands; 4 Department of Medical Oncology, Haga Hospital, Den Haag, The Netherlands; 5 Department of Medical Psychology, Leiden University Medical Center, Leiden, The Netherlands

**Keywords:** Scalp cooling, Alopecia, Paclitaxel, Randomized controlled trial, Chemotherapy-induced alopecia distress scale (CADS)

## Abstract

**Purpose:**

Scalp cooling can prevent chemotherapy-induced alopecia (CIA). Previously, the post-infusion cooling time (PICT) could be successfully reduced in docetaxel-treated patients from 90 to 45 and 20 min. Therefore, it seems plausible that the PICT can be shortened for paclitaxel-treated patients as well.

**Methods:**

Patients treated with weekly paclitaxel were included in this multi-centre trial and randomly assigned to a PICT of 45 or 20 min. The results were compared to a standard PICT of 90 min, derived from prospective collected data from the Dutch Scalp Cooling Registry. The primary endpoint was the percentage of patients who decide to not wear a wig or head covering. Secondary endpoints were the degree of CIA assessed with the Dean scale for assessment of hair loss; alopecia graded according to NCI CTC toxicity version 4.03 (CTCAE4.03); tolerance of scalp cooling and perceived distress of CIA.

**Results:**

Ninety-one patients were enrolled in this study; 74 patients were evaluable for hair loss. Hair preservation was successful in 27 patients (75%) with a PICT of 45 min and in 31 patients (82%) with a PICT of 20 min. There was no difference in success rate with the standard PICT of 90 min (85%, *p* = 0.29). Similar success rates were seen when using the Dean scale and CTCAE assessment, with no differences between groups (*p* = 0.12 and *p* = 0.38).

**Conclusions:**

A 20 min PICT is as effective as 45 and 90 min to prevent weekly paclitaxel-induced alopecia and should be the new standard of care.

**Trial register:**

ClinicalTrials.gov Identifier: NCT03266185.

## Introduction

Chemotherapy-induced alopecia (CIA) is one of the most distressing side effects of systemic cancer treatment and has a negative impact on the wellbeing of many patients with cancer [1]. The incidence of severe CIA is less often observed in patients treated with novel agents like targeted therapies, checkpoint inhibitors, and oral chemotherapy; although in some situations, these agents are combined with cytotoxic drugs that often cause CIA [2]. Therefore, prevention of CIA remains an important topic of supportive care.

Scalp cooling proved to be the most effective method to prevent CIA; it can prevent or minimise CIA in approximately half of all patients, depending on many factors, such as type and dosage of chemotherapy [3–6]. The mechanism of scalp cooling to prevent CIA is by inducing local vasoconstriction [7]. Vasoconstriction reduces blood flow to the hair follicles by interrupting the uptake of cytotoxic agents, making hair follicles less vulnerable to the damage caused by chemotherapy. Treatment with scalp cooling has been shown to be safe and well tolerated. Previous research showed no difference in overall mortality between scalp-cooled and non-scalp-cooled patients with breast cancer, and no increased risk of developing scalp skin metastasis [8–11]. The most reported side-effect of scalp cooling is headache, mostly mild, and generally well treated with paracetamol (acetaminophen) [12].

The Dutch Scalp Cooling Registry has collected data of > 7000 patients since 2006 [13]. The majority of patients were treated for breast cancer and in a (neo)adjuvant setting. More than half of these patients did decide not to wear a head cover at the last scalp cooling cycle. High rates of success have been particularly seen in patients treated with taxanes, up to 80–90% [3].

Scarce information is available about optimal scalp cooling techniques. One of the factors that could be of influence is the cooling time. The optimal pre-cooling time is established to be around 30 min, since the temperature of the scalp then hardly decreases anymore. Regarding the post-infusion cooling time (PICT), there is more uncertainty. In previous published research, most PICT ranged from 15 min to 4 h [14]. In the Netherlands, a PICT of 90 min is mostly used as a standard care in daily practice. In theory, the cooling period after administration of cytotoxic agents should be related to their pharmacokinetics and based on calculations of half-life times of the drugs and their active metabolites in the plasma. However, there is a great patient variability in plasma half-life times and bioavailability of cytotoxic agents. Combination chemotherapy might change pharmacokinetic profiles and duration of infusion and subsequent peaks of cytotoxic agents differ [15]. In the past, there was a general trend in favour of a longer PICT; however, shortening the PICT may lead to a decreased exposure time of hair follicles to toxic drugs. Furthermore, repair mechanism of hair root cells might be inhibited for a longer period if there is an unnecessary long PICT.

Previous research has shown very good results of scalp cooling in docetaxel-treated patients when shortening the PICT from the standard of 90 min to 45 and 20 min [12, 15]. Whether the PICT can be safely shortened in the administration of other chemotherapeutic agents has not been studied so far. It seems plausible that the PICT can be shortened for paclitaxel-treated patients as well, at least at weekly dosages of 70–90 mg/m^2^, as results of scalp cooling are very good for that regimen [3]. Paclitaxel and docetaxel, both classical taxanes, share similar mechanisms of action and comparable plasma terminal half-life times; between 8 and 12 h in paclitaxel and 12 h in docetaxel [16].

The present prospective randomized multi-centre study is aimed to assess the efficacy of scalp cooling in patients treated with paclitaxel-containing chemotherapy with a 45- and 20-min PICT, in comparison with the standard 90 min derived from the Dutch prospective registry. A shorter PICT would be an advantage for both the patient, who can spend less time in the hospital, as well as for the logistic and financial burden at oncological departments.

## Methods

### Study design

The study is a prospective, open-label, randomized multi-center trial, in which patients treated with 70–90 mg/m^2^ paclitaxel were randomly assigned (1:1 ratio) to a 45- or 20-min PICT. The results from a PICT of 45 and 20 min were compared with the standard PICT of 90 min, using previous collected data from the Dutch Scalp Cooling Registry. All procedures performed were in accordance with the ethical standards of the institutional and/or national research committee and with the 1964 Helsinki declaration and its later amendments or comparable ethical standards. The primary study outcome was efficacy of scalp cooling with a 45- and 20-min PICT, in comparison with the standard 90 min, defined by the patient’s self-determined need to wear a wig or other head covering. Patients were considered evaluable for the primary endpoint if they had received at least three administrations of chemotherapy or if they discontinued scalp cooling before that time due to severe hair loss. Secondary study outcome measures was difference in both study arms between the following outcomes: the degree of CIA assessed with the Dean scale for hair loss [17]; the grade of alopecia according to the National Cancer Institute Common Terminology Criteria for Adverse Events (NCI CTCAE), version 4.03; Tolerance of scalp cooling, assessed by a (self-defined) visual analogue scale (VAS) of 0–10, in which 0 represent “not tolerable at all” and 10 means “very tolerable”, at every scalp cooling session. Perceived distress of CIA (descriptive) was assessed with the chemotherapy-induced alopecia distress scale (CADS) questionnaire (1) before the start of chemotherapy (baseline), at cycle 3 and at cycle 6.

### Patients

Patients were recruited from the Leiden University Medical Center, Leiden, the Dutch Cancer Institute, Amsterdam, and The Haga Hospital, the Hague, before the start of paclitaxel-containing chemotherapy. Written informed consent was obtained from all patients included in the study. Eligibility criteria were patients receiving weekly administered paclitaxel-containing chemotherapy (minimal 3 planned administrations) in a dose of 70–90 mg/m^2^, age ≥ 18 years, a WHO performance status 0–2 and a life expectancy of ≥ 3 months. Exclusion criteria were treatment with paclitaxel in sequential schemes (for example after doxorubicin, cyclophosphamide (AC)), alopecia before the start of treatment and rare cold-related disorders like cold sensitivity, cold agglutinin disease, cryoglobulinaemia, cryofibrinogenaemia, and cold posttraumatic dystrophy.

### Scalp cooling

All participating hospitals used Paxman cooling machines for one person (PSC-1). Scalp cooling was applied 30 min prior to the chemotherapy administration until 20 or 45 min after the end of infusion. Paclitaxel was infused over 1 h in all patients. Scalp cooling was applied during all planned cycles of chemotherapy, unless the patient decided to stop the cooling procedure based on intolerance, hair loss, or for other reasons.

### Statistical methods

Previous research showed an approximately success rate of 85% for scalp cooling in patients treated with monotherapy weekly paclitaxel [3]. Power analysis was based to demonstrate a 30% difference in requiring a wig or other head covering between groups. This can be detected with 45 patients in each randomized group with 80% power and a two-sided alpha value of 0.05, assuming that approximately 1/5 of patients will not be evaluable [12, 15]. The proportion of patients who need to wear a wig or other head covering was summarized by descriptive statistics and tested using Pearson’s Chi-squared test, as well as the Dean scale and the NCI CTCAE v4.03. The analyses were carried out on all patients on an intention-to-treat (ITT) basis. The VAS and CADS were tested using the Mann–Whitney *U* test. SPSS 25.0 was used for all analyses. All tests of significance were two-sided and considered statistically significant when *p* < 0.05.

## Results

### Patients and treatment

**Fig. 1:**
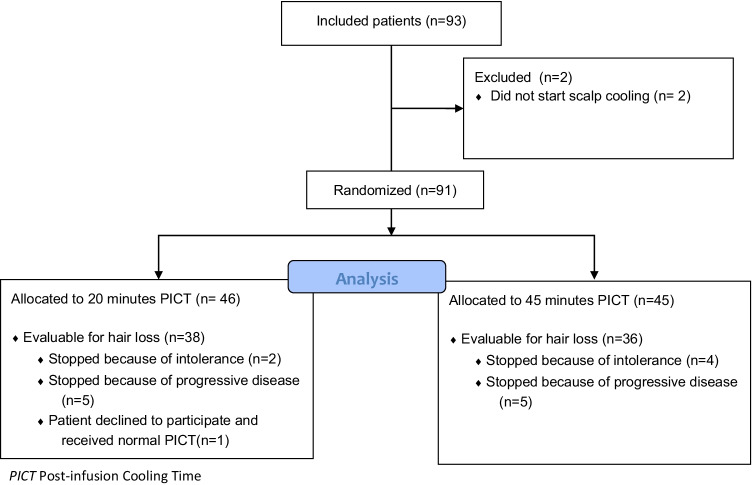
Consort flow diagram. PICT, post-infusion cooling time

Between January 2018 and August 2020 a total of 93 patients were included in this trial. Ninety-one patients were randomized between a PICT of 45 or 20 min. Two patients decided not to start with scalp cooling (Fig. 1). Patients’ characteristics are summarized in Table 1. The median age was 57 years, 86 (95%) patients were female, and most patients were treated for breast cancer. There were no differences between patients’ characteristics in the 45- and 20-min group and patients from the Dutch Scalp Cooling Registry. Patients received a mean of 11 (minimum 1–maximum 32) weekly cycles paclitaxel.

**Table 1:** Patient characteristics

	20-min PICT (*n* = 46)	45-min PICT (*n* = 45)	90-min PICT (*n* = 723)
Age (mean, SD)	57 (11.1)	58 (13.7)	57 (12.1)
Gender Female	42 (91%)	44 (98%)	723 (97%)
Cancer Breast Ovarian Endometrial Lung Other Paclitaxel dosage 70 mg/m^2^ 80 mg/m^2^ 90 mg/m^2^	29 (63%) 7 (15%) 3 (7%) 4 (9%) 3 (7%) 1 (2%) 39 (85%) 6 (13%)	33 (73%) 5 (11%) 2 (4%) 2 (4%) 3 (7%) 1 (2%) 41 (91%) 3 (7%)	596 (82%) 67 (9%) 6 (1%) 2 (< 1%) 52 (7%) 3 (< 1%) 572 (79%) 148 (20%)
Treatment Paclitaxel monotherapy Combination chemotherapy Combination monoclonal antibodies Combination chemotherapy + monoclonal antibodies Setting (Neo) adjuvant Palliative	14 (30%) 6 (13%) 13 (28%) 12 (26%) 21 (46%) 25 (54%)	14 (30%) 8 (17%) 13 (28%) 11 (24%) 24 (53%) 21 (47%)	505 (70%) 106 (15%) 105 (15%) 7 (1%) 215 (30%) 503 (70%)
Number of cycles with scalp-cooling (mean, SD)	11.0 (6.8)	10.0 (5.4)	10.0 (5.5)

*PICT* Post-infusion cooling time, *SD* standard deviation

^*^Frequencies (%), some percentages may not total 100 because of rounding

### Efficacy

A total of 74 out of 91 included patients were evaluable for hair loss. Seventeen patients could not be evaluated (Fig. 1): 10 patients received less than 3 cycles of chemotherapy (due to tumour progression or paclitaxel toxicity), 6 patients stopped scalp cooling because of intolerance during the first three treatments, and 1 patient decided that she wanted the normal PICT of 90 min after randomization. There were no differences in the need to wear head covering between the groups with 45- and 20 min PICT and the control group of 90-min PICT. There neither were differences in the Dean scale and the NCI CTCAE grade between 45- and 20-min PICT (Table 2).

**Table 2:** Results of scalp cooling in patients evaluable for hair loss

	20-min PICT (*n* = 38)	45-min PICT (*n* = 36)	90-min PICT(*n* = 723, Dutch Scalp Cooling Registry)	*P* value
Primary endpoint (self-determined need to wear head covering)				
Patients with head covering	7 (18.4%)	9 (25.0%)	112 (15.5%)	
Patients without head covering	31 (81.6%)	27 (75%)	611 (84.5%)	0.29
Secondary endpoints	(*n* = 26)	(*n* = 26)		
Dean scale for Alopecia (mean, SD) Score 0–2 Score 3–4	1.31 (1.01) 21 (80.8%) 6 (19.2%)	1.77 (1.07) 21 (80.8%) 5 (19.2%)	n/a	0.12 1.0
NCI CTCAE grade of alopecia (mean, SD) Score 0–1 Score 2	1.00 (0.63) 21 (80.8%) 5 (19.2%)	1.15 (0.61) 21 (80.8%) 5 (19.2%)	n/a	0.38 1.0

*PICT* Post-infusion cooling time, *NCI CTCAE* National Cancer Institute Common Terminology Criteria for Adverse Events

### Tolerability

Scalp cooling in both randomised groups was well tolerated with a median VAS-score of 8.1 (range 1–10) in 527 cooling sessions. There were no differences in tolerability between 45- or 20-min PICT (8.4 versus 8.5 in 20 respectively). Six out of 91 patients (6.6%) stopped scalp cooling because of intolerance, 4 patients in the 45-min group (8.9%), and 2 patients in the 20-min group (4.3%).

### Distress

**Fig. 2:**
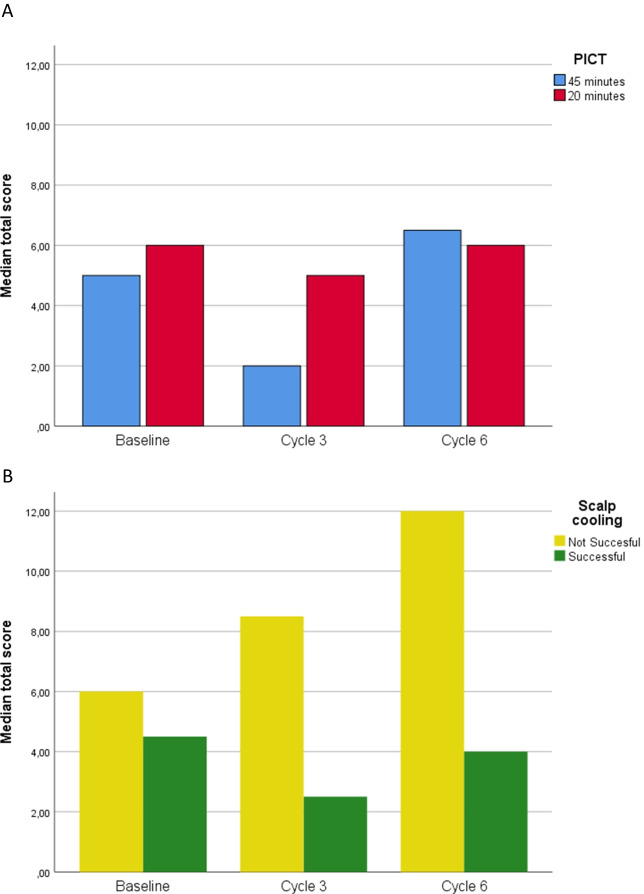
Severity of CIA distress measured with the CADS questionnaire. **A** Differences between 45- and 20-min PICT. **B** Differences between successful and not successful scalp cooling. CIA, Chemotherapy-induced alopecia; CADS, chemotherapy-induced alopecia distress scale; PICT, post-infusion cooling time

No differences in the median CADS scores, or proportion of patients that perceived high distress (score of 14 or above) were found between patients randomized to 45- or 20-min PICT. However, for patients with unsuccessful scalp cooling, we did find a significant increase in the median scores, when compared to the patients with successful prevention of hair loss at cycle 3 and 6 (*p* =  < 0.01, Table 3, Fig. 2).

**Table 3:** Severity of CIA distress measured with the CADS questionnaire

	45-min PICT	20-min PICT	*p* value
Baseline			
Median total score (range)	5.0 (0–23)	4.0 (0–20)	0.80
Low distress (*n* (%))	23 (92.0)	25 (92.6)	0.94
High distress (*n* (%))	2 (8.0)	2 (7.4)	
Cycle 3			
Median total score (range)	2.0 (0–21)	5.0 (0–19)	0.31
Low distress (*n* (%))	17 (89.5)	18 (85.7)	0.85
High distress (*n* (%))	2 (10.5)	3 (14.3)	
Cycle 6			
Median total score (range)	6.5 (0–25)	6.0 (0–23)	0.81
Low distress (*n* (%))	16 (88.9)	16 (88.9)	1.00
High distress (*n* (%))	2 (11.1)	2 (11.1)	
	Successful scalp cooling *n* (%)	Unsuccessful scalp cooling* n* (%)	*p* value
Baseline			
Median total score (range)	4.5 (0–20)	6 (0–23)	0.39
Low distress (*n* (%))	29 (90.6)	11 (91.7)	
High distress (*n* (%))	3 (9.4)	1 (8.3)	0.97
Cycle 3			
Median total score (range)	2.5 (0–17)	8.5 (0–21)	< 0.01
Low distress (*n* (%))	29 (96.7)	6 (60.0)	
High distress (*n* (%))	1 (3.3)	4 (40.0)	0.09
Cycle 6			
Median total score (range)	4.0 (0–14)	12 (2–25)	< 0.01
Low distress (*n* (%))	26 (96.3)	6 (66.7)	
High distress (*n* (%))	1 (3.7)	3 (33.3)	0.19

The CADS’ total score ranges from 0 to 51, low distress: 0–13 score, high distress: score of 14 or above

*CIA* Chemotherapy-induced alopecia, *CADS* chemotherapy-induced alopecia distress scale, *PICT* post-infusion cooling time

## Discussion

This study is the first to show that the PICT can be decreased to 20 min for patients treated with weekly paclitaxel (dosage of 70–90 mg/m^2^). No difference in the need to wear head covering was found between 45- and 20-min PICT. In addition, the results of a shorter PICT were comparable with the efficacy of the previous collected data from the Dutch Scalp Cooling Registry with a standard 90-min PICT. These clinical practise changing results were seen in both paclitaxel monotherapy, as well as in paclitaxel regimens combined with other chemotherapy or monoclonal antibodies.

In this study, 78% of the patients felt they did not need head covering after being treated with weekly paclitaxel. Similar to previous trials, the number of patients that did not need head covering was high. Scalp cooling was well tolerated with high median VAS scores. Our results are in accordance with the results of the other scalp cooling studies with a shorter PICT in 3 weekly docetaxel-containing chemotherapy [12, 15] and results of scalp cooling in taxane-based chemotherapy [3, 6, 18]. In the docetaxel PICT study 61% of the patients were male, whereas in our study predominantly female patients (95%) participated. In the study from Komen et al., 95% of the male patients was not wearing any head covering during the trial, in contrast to the female patients, where only 41% did not need head covering [12]. There is a general assumption that men are less likely to wear a wig or other head covering and accept more hair loss. Nevertheless, in our study with mostly women, the results are still excellent and comparable.

Consistent improvement of patient’s QoL when using scalp cooling has not been reported so far [19]. There are several possible explanations for the lack of positive impact. Firstly, QoL assessments are generally covering a variety of domains like global health status, physical, and emotional symptoms, reflecting little information on well-being specially associated with CIA. The CADS, used in this study, will be more appropriate to assess the impact of CIA on QoL in patients who use scalp cooling. Secondly, patients who develop CIA despite scalp cooling can have worse QoL, when compared to patients who accept hair loss from early on and choose not to undergo scalp cooling [20]. In our study, we did observe significantly increasing CADS scores in patients with hair loss despite scalp cooling, consistent with this assumption. A recent study indicates that impact on body image and signs of depression are related to patients´ expectations of the efficacy of scalp cooling [21]. Additional, coping with hair loss can negatively impact patients’ lives [22]. Therefore, careful counselling and guidance, with setting of realistic expectations, for patients who choose scalp cooling is of importance. Increasingly, the incidence of permanent chemotherapy-induced alopecia (pCIA) after taxane chemotherapy is reported [23, 24]. A phenomenon, especially seen after docetaxel treatment; however, it has been described after paclitaxel treatment as well [24]. Although pCIA is rare, this emphasizes the importance of offering scalp cooling when initiating taxane treatment, as it has shown to diminish the incidence of pCIA [24].

Spending 70 min less in the hospital during every administration of paclitaxel as a result of a shorter PICT is a great benefit for patients as some of them reject scalp cooling because of the time investment [25]. Furthermore, it proved to be feasible for patients treated with taxanes. The advantage of an early discharge, which creates opportunities to treat more patients on one day, is desirable and cost-effective for hospitals. Future research should explore if a shorter PICT should be the standard for more types of chemotherapy.

In conclusion, this study showed retainment of very good results of scalp cooling in weekly paclitaxel-containing chemotherapy regimens, despite shortening the PICT. Based on this study, a 20-min PICT can be recommended as the new standard PICT for patients treated with weekly paclitaxel-containing schedules.

## Data Availability

The authors confirm they have full control of all primary data and agree to allow the journal to review their data if requested. The datasets generated and/or analyzed during the current study are not publicly available as sharing is not explicitly covered by patient consent.
